# Effect of Ionizing Radiation on the Bacterial and Fungal Endophytes of the Halophytic Plant *Kalidium schrenkianum*

**DOI:** 10.3390/microorganisms9051050

**Published:** 2021-05-13

**Authors:** Jing Zhu, Xiang Sun, Zhi-Dong Zhang, Qi-Yong Tang, Mei-Ying Gu, Li-Juan Zhang, Min Hou, Amir Sharon, Hong-Li Yuan

**Affiliations:** 1 State Key Laboratory of Agrobiotechnology and Key Laboratory of Soil Microbiology, Ministry of Agriculture, College of Biological Sciences, China Agricultural University, Beijing 100193, China; zhujing2020@hotmail.com; 2 Institute of Applied Microbiology, Xinjiang Academy of Agricultural Sciences/Xinjiang Key Laboratory of Special Environmental Microbiology, Urumqi 830091, China; zhangzheedong@sohu.com (Z.-D.Z.); tqy25@163.com (Q.-Y.T.); gmyxj2008@163.com (M.-Y.G.); zhanglijuan@xaas.ac.cn (L.-J.Z.); hmde_092@163.com (M.H.); 3 School of Life Sciences, Hebei University, Baoding 071002, China; 4 School of Plant Sciences and Food Security, Faculty of Life Sciences, Tel Aviv University, Tel Aviv 69978, Israel; amirsh@tauex.tau.ac.il

**Keywords:** radiobiology, microbial ecology, endophyte, stressed environment, halophyte

## Abstract

Endophytic bacteria and fungi colonize plants that grow in various types of terrestrial and aquatic ecosystems. Our study investigates the communities of endophytic bacteria and fungi of halophyte *Kalidium schrenkianum* growing in stressed habitats with ionizing radiation. The geochemical factors and radiation (at low, medium, high level and control) both affected the structure of endophytic communities. The bacterial class Actinobacteria and the fungal class Dothideomycetes predominated the endophytic communities of *K. schrenkianum*. Aerial tissues of *K. schrenkianum* had higher fungal diversity, while roots had higher bacterial diversity. Radiation had no significant effect on the abundance of bacterial classes. Soil pH, total nitrogen, and organic matter showed significant effects on the diversity of root endophytes. Radiation affected bacterial and fungal community structure in roots but not in aerial tissues, and had a strong effect on fungal co-occurrence networks. Overall, the genetic diversity of both endophytic bacteria and fungi was higher in radioactive environments, however negative correlations were found between endophytic bacteria and fungi in the plant. The genetic diversity of both endophytic bacteria and fungi was higher in radioactive environments. Our findings suggest that radiation affects root endophytes, and that the endophytes associated with aerial tissues and roots of *K. schrenkianum* follow different mechanisms for community assembly and different paradigms in stress response.

## 1. Introduction

Endophytes are symbiotic microbes that live within a plant and are important components of the plant microbiome [1]. These microbes are found distributed in terrestrial and marine plants, and have high phylogenetic diversity and ecological functions. Endophytes and their host plants have coevolved, and both benefit from this mutual symbiosis. Studies have shown that endophytes improve host plant physiology to adapt to stressed environments; endophytes reprogrammed the host response to pathogen invasion and increased toxic chemical production to provide protection against herbivores [2,3,4]. In the other side, endophytes live inside plant tissues, which protect them from dehydration, poor nutrition, ultraviolet radiation, and competition [5,6]. The mutual interaction profoundly shaped communities of both plant and microbes and changed the biodiversity, but the related knowledge remains limited. Therefore, the biology and ecology of endophytic microbiota and their hosts have gained attention and become important research topics.

The endophytic microbial community structure is influenced by various biotic and abiotic factors, such as host identity, climate, biological or circumstantial stresses, and geochemical factors [7]. Host identity is the major factor that determines endophytic community composition [7,8,9,10]. Whereas, environmental factors have a complex influence on the endophytic community. Studies have shown that the endophytic population is affected by climate changes such as increased carbon dioxide emission, global warming, and drought [11]. Rodriguez, Henson, Volkenburgh, Hoy, Wright, Beckwith, Kim and Redman [2] demonstrated habitat-specific, symbiotically-conferred stress tolerance in plants under high-stress environment. Therefore, research on how endophytic community shift under stressed conditions will shed light into fundamental issues in understanding how the mutual interaction between plants and endophytes promotes the adaption of the symbiotic entity.

Ionizing radiation is a type of radiation, in the form of electromagnetic waves or particles, with sufficient energy to ionize an atom or a molecule. Overdosed exposure to ionizing radiation causes harmful effects in living organisms. Ionizing radiation is generally detected in areas exposed to radioactive minerals, in high altitude environments, or in aerospace. Nuclear power plants, nuclear weapon tests, nuclear accidents, and mining produced new radioactive habitats. However, certain plants, animals, and microbes survive under radioactive environment. Studies have reported the existence of shrubs, rodents, terrestrial algae, and fungi in Nevada Test Site (NTS) few years after the nuclear tests [12,13,14,15,16]. Durrell and Shields [17] isolated 41 fungal taxa from soil within a mile radius of ground zero sites two years after nuclear tests in NTS. Thirty-seven culturable fungal species of 19 genera were detected on the walls and other building structures in the inner parts of the shelter of the damaged fourth unit of the Chernobyl Nuclear Power Plant [18]. Radiation has also resulted in shifts in the local microbial communities. For example, the soil fungal community structure appeared to shift toward species that may be more radiation resistant, and melanin-containing fungi ascended to dominate the soil fungal communities with increase in radionuclide pollution [19]. Lavrinienko, et al. [20] correlated with the gut microbiomes of bank vole *Myodes glareolus* inhabiting the Chernobyl zone with the radioactivity level. Wehrden et al. observed physiological and morphological changes in the organisms of radioactive environments and other negative effects of radiation on the ecosystem [21]. The biological and ecological traits of ecosystem under radiation stress is a great concern in policy making on nuclear power utilization and in designing aerospace sustainable life support system. However, there are no studies on the symbiotic microbes of plants exposed to ionizing radiation.

Endophytic microbes symbiotically associate with host plants, and the symbiotic entity of inter-kingdom jointly challenge harsh circumstances. However, the relationship between endophytes and the host plant under radioactive environment is unknown. High-radiation habitats because of Caesium-137 (^137^Cs) accumulation from historic nuclear test exist in arid, saline semi-arid desert of northwest China. This paper investigates the endophytic microbes (fungi and bacteria) in the roots and aerial parts of Amaranthaceae halophyte *Kalidium schrenkianum* Bunge. ex Ung.-Sternb., a dominant population of local flora. Here, we collected plant samples and soil samples from sites representing different levels of radiations. We aim to understand the diversity and community structure of endophytic bacteria and fungi in a radioactive habitat; determine the main environmental factor (geochemical characteristics of soil and local radiation level) shaping the endophytic communities; and elucidate the shift in pattern of endophytic microbes with radiation level.

## 2. Materials and Methods

### 2.1. Study Site and Sampling

This study was conducted in Hoxud County in the Xinjiang Uyghur Autonomous Region of China (91°45′42″ E, 40°39′75″ N). This region experiences a semi-arid climate with a mean annual temperature of 12.56 °C and a mean annual precipitation of 591 mm. The sampling sites are located in the watershed area of seasonal floods from radiation-contaminated region with ^137^Cs accumulation. The soil had a salt content above 2% in the surface layer (< 20 cm deep) and a radionuclide level 3–5 fold that of normal soil. *K. schrenkianum* was the dominant population of the local habitat.

Plant materials were collected from four sites with different environmental radioactivity levels during August and September 2017 (Table 1). Plant materials were collected using a random sampling approach from a 50 × 50 m^2^ square plot in each site. Five plants were randomly selected (at least 15 m apart from each other) and uprooted from each square plot. Whole plants were placed in large autoclaved paper envelopes, labelled, transported to laboratory in an icebox, and stored at −80 °C until further process. In addition, five soil samples were collected with shovels from the surface soil (0–20 cm deep) along the diagonal of the square plot. Soil samples from each site were sieved to remove rocks and plant litter, thoroughly mixed, packed and labelled in cloth bags, and stored at 4 °C to transport to the laboratory.

### 2.2. Analysis of Soil Physical and Chemical Properties

The radioactivity of ^137^Cs in soil samples was detected in Northwest Institute of Nuclear Technology (Xi’an, China). One hundreds grams of the soil samples were put into an environmental source box (ϕ 45 cm × 25 cm plastic box), and was measured on an HPGe γ spectrometer (DSPEC-281, ORTEC, Tennessee, TN, USA). The sample was placed 8 cm away from the detector, and the measurement time ranged from 1–3 d. The 661 keV peak of ^137^Cs in soil samples was measured and analyzed by net counting, and the radionuclide activity of ^137^Cs in the soil was calculated [22]. The soil samples were divided into four radiation levels. Level Control (CK) were the soil samples from area without radiation pollution (10–20 Bq/kg), while the level Low (L, 20–40 Bq/kg), Medium (M, 40–60 Bq/kg), and High (H, >60 Bq/kg) were sampled in areas of light, moderate, and high radionuclide contamination, respectively.

The mixed soil samples from each site (of different radiation levels) were proceeded to detected the physical and chemical indicators, such as pH, electrical conductivity (EC), organic matter (OM), total nitrogen (TN), soluble nitrogen (SN), available phosphorus (P), available potassium (K), salt (Sal), chloride ion (Cl), sulfate (Sulf), calcium ion (Ca), magnesium ion (Mg), and sodium ion (Na), were measured in the Institute of Quality Standards & Testing Technology for Agro-Products, Xinjiang Academy of Agricultural Sciences.

### 2.3. DNA Extraction, PCR, and NGS Sequencing

The aerial parts and roots of *K*. *schrenkianum* were separately conducted to DNA extraction, and altogether 40 samples (5 plant replicates × 4 radiation levels × 2 tissue types) were used for endophyte analysis. Each plant material (20 g) was surface sterilized with 75% (*v/v*) ethanol for 1 min, 3.25% (*w/v*) sodium hypochlorite for 3 min, and 75% (*v/v*) ethanol for 30 s [23]. Genomic DNA was extracted following CTAB method [23]. One gram of the surface-sterilized plant material was freeze-dried using liquid nitrogen, homogenized with a mortar and pestle, transferred to a tube with 5 mL 2× cetyltrimethylammonium bromide (CTAB) extraction buffer (2% (*w/v*) CTAB, 100 mM Tris-HCl, 1.4 M NaCl, 20 mM EDTA, 1.5% (*w/v*) polyvinyl-pyrolidone (PVP), 0.5% (*w/v*) 2-mercaptoethanol; pH 8.0; preheated to 65 °C), and then incubated in a 60 °C water bath for 30 min with occasional gentle swirling. 500 µL of chloroform:isoamyl alcohol (24:1) was added into each tube and mixed thoroughly to form an emulsion. The mixture was spun at 11,900× *g* for 15 min at room temperature and the aqueous phase containing DNA was removed into a fresh 1.5 mL tube and reextracted for two more times. Then, 50 µL of 5 M KOAc was added into the aqueous phase followed by 400 µL of isopropanol and inverted gently to mix. The genomic DNA was precipitated overnight at 4 °C and then spun at 9200× *g* for 2 min. The DNA pellet was washed with 70% (*v/v*) ethanol twice and dried using SpeedVac2 (AES 1010; Savant, Holbrook, NY, USA) for 10 min or until dry. The DNA pellet was then resuspended in 100 µL TE buffer (10 mM Tris-HCl, 1 mM EDTA). The concentration of DNA was measured using a NanoDrop 1000 Spectrophotometer (Thermo Scientific, Wilmington, NC, USA).

The V5-V7 hypervariable region of the bacterial 16S ribosomal RNA gene was amplified using 799F (AACMGGATTAGATACCCKG) [24] and 1193R (ACGTCATCCCCACCTTCC) primers [25]. The fungal internal transcribed spacer region 1 (ITS1 region) of ribosomal RNA was amplified using ITS1F (CTTGGTCATTTAGAGGAAGTAA) [26] and ITS2 (GCTGCGTTCTTCATCGATGC) primers [27]. All PCR reactions were carried out in 30 µL reactions with 15 µL of Phusion^®^ High-Fidelity PCR Master Mix (New England Biolabs, Ipswich, UK); 0.2 µM of forward and reverse primers, and about 10 ng template DNA. Thermal cycling consisted of initial denaturation at 98 °C for 1 min, 30 cycles of denaturation at 98 °C for 10 s, annealing at 50 °C for 30 s, and elongation at 72 °C for 30 s, followed by a final extension at 72 °C for 5 min. Mix same volume of 1× loading buffer (contained SYB green) with PCR products and operate electrophoresis on 2% agarose gel for detection. Then, PCR products was purified with GeneJETTM Gel Extraction Kit (Thermo Scientific, Waltham, MA, USA). Sequencing libraries were generated using Ion Plus Fragment Library Kit 48 rxns (Thermo Scientific, Waltham, MA, USA) following manufacturer’s recommendations. The library quality was assessed on the Qubit@ 2.0 Fluorometer (Thermo Scientific, Waltham, MA, USA). At last, the library was sequenced on an Ion S5TM XL platform.

### 2.4. Bioinformatic Analysis

Raw reads from bacterial and fungal dataset were demultiplexed and quality filtered using QIIME (version 1.7.0). Reads with a quality score <20 and those lacking complete barcode and primers were excluded from further analysis. Chimeric sequences were removed using 32-bit USEARCH (version 11; Edgar, R.C.; 2018). Subsequently, DADA2 workflow [28] was used to remove singletons and doubletons. Both bacterial and fungal datasets were dereplicated to generate the amplicon sequence variants (ASVs).

Taxonomy was assigned to bacterial and fungal ASVs using Naïve Bayes approach with minimum 75 bootstrap calls following DADA2 workflow [28] against SILVA version 132 [29] and UNITE general FASTA release for Fungi version 8.0 [30], respectively. For bacterial dataset, those ASVs that were not assigned to bacterial genus, were clustered into different operational taxonomic units (OTUs) based on 97% similarity with function *otu* in “kmer” package [31]. One random sequence was selected from each OTU, and assigned based on SILVA references following the above method. Then, the taxonomy assignments of bacterial ASVs and OTUs were combined to an overall bacterial taxon-sample table. All ASVs and OTUs that were assigned to non-bacteria, Cyanobacteria phylum, or Rickettsiales order were removed from the overall taxon-sample table. The ASVs or OTUs in the overall table were then agglomerated at the bacterial genus level with identical assignments using “phyloseq” package as described in DADA2 workflow [28]. For fungal dataset, those ASVs that were not assigned to fungal species, were similarly clustered, identified based on UNITE references for eukaryotes version 8.0 [30], generated fungal taxon-sample dataset, filtered off non-fungal taxa, and agglomerated fungal taxa at species level with DADA2 workflow mentioned above. Please see supplementary file 1 and Figure S1 for more details and schematic diagram.

The ASV and OTU sequences have been deposited in GenBank of National Center for Biotechnology Information under the accession numbers PRJNA613570 (bacterial 16S sequences) and MT351182-MT353643 (fungal ITS1 sequences) and the raw sequences in the Sequence Read Archive of NCBI under BioProject PRJNA625640 (for bacterial data) and PRJNA613597 (for fungal data).

### 2.5. Data Analysis

Read counts from all samples were of the same order of magnitude (18,834 to 44,787 for bacterial dataset and 9726 to 62,613 for fungal dataset). Singletons, and doubletons were first filtered out from both bacterial and fungal datasets. To decrease the noise, taxa with the sum of relative abundance less than 0.001 were removed. This resulted in a core dataset of 693 taxa by 40 samples in bacterial dataset and 364 taxa by 40 samples in fungal dataset. The raw taxa counts were normalized to abundance using Hellinger transformation.

Statistical analyses were performed using R (version 3.6.1) [32]. Graphs were plotted with R packages “ggplot2” [33], “grid” [34], and “gridExtra” [35]. Two-way analysis of variance (two-way ANOVA) was carried out to test the effect of plant tissue type or radiation level on the richness and diversity of bacterial and fungal microbiota with function *aov* in “stats” package [32]. Type 1 error rates had a Benjamini-Hochberg (FDR) *p* value correction performed for ANOVA models with function *p.adjust* in “stats” package [32,36,37]. Significant differences between the microbial populations were further compared using Tukey’s honestly significant difference (HSD) test with function *HSD.test* in “agricolae” package [38].

The distance matrices of community composition (Hellinger-transformed OTU read data) of endophytic fungi were constructed by calculating dissimilarities using Bray–Curtis method [39]. Non-metric multidimensional scaling (NMDS) was used to visualize the community composition dissimilarity of endophytic bacteria or fungi among the different plant tissues or radiation levels using *metaMDS* function in “vegan” package [40]. Environmental factors were fit to NMDS with Bray–Curtis distance using *envfit* function in “vegan” package. Analysis of similarities (ANOSIM) was applied to statistically test the significant differences in microbial composition between plant tissues or among radiation levels. Permutational multivariate analysis of variance (PerMANOVA) with 999 permutations was implemented with *adonis* in “vegan” package to investigate the environmental influence on microbiota composition.

The effect of different environmental factors (explanatory variables) on endophyte abundance or richness (genus level for bacteria and species level for fungi) was tested using Poisson generalized linear models (GLM) with stepwise selection by AIC. This analysis was performed using function *glm* in “stats” package and function *stepAIC* in “MASS” package [32,36,41]. The data distribution was tested with function *shapiro.test* in “stats” package. All data were calculated with Poisson distribution and overdispersion in data was tested with function *qcc.overdispersion.test* in “qcc” package [42]. Type 1 error rates were FDR-corrected with the method mentioned above.

Co-occurrence analysis was applied for genera and unidentified OTUs of endophytic bacteria and fungi. The bacterial dataset was the same dataset of 693 taxa by 40 samples described above. The fungal dataset of 364 fungal taxa by 40 samples generated in 2.5 with raw taxa counts were agglomerated at genus level as mentioned above, and then Hellinger transformed. The interactions between endophytic bacteria and fungi were inspected in aerial tissues and in roots, with combined bacterial and fungal datasets. In addition, the co-occurrence analyses were separately conducted for bacterial and fungal communities in aerial tissues or roots at each radiation levels. Spearman’s *rho* statistic is used to estimate correlation with function *cor.test* in “stats” package [32]. The co-occurrence networks were visualized with “igraph” package [43]. Network characteristics were determined using functions in “bipartite” package [44].

Intra-genus genetic diversity of bacteria and fungi from control and three treatment levels were evaluated by computing the pairwise distances of DNA sequences within the groups. Only the ASVs assigned taxonomy at the genus level for bacteria and fungi were included in the analysis. Pairwise distance was calculated among all ASVs available in a certain genus from one treatment level using “K80” model with *dist.dna* in “ape” package [45]. One-way ANOVA was applied to test the difference significance of intra-genus genetic diversity, as well as all sequence distances regardless of genera, among four treatments.

## 3. Results

### 3.1. General Description

The Ion platform produced approximately 2,790,479 raw reads for prokaryotes from 40 samples by sequencing V5-V7 hypervariable region of the bacterial 16S ribosomal RNA gene. After quality control, denoising, and removal of chimera sequences, 1,235,434 high-quality sequences were obtained. A total of 19,367 ASVs recovered in the final dataset were subjected to taxonomy assignment; 8788 ASVs were assigned to 317 genera. Meanwhile, 10,579 ASVs, which could not be identified at the genus level, were clustered into 2002 OTUs at 97% identity level and were again subjected to taxonomy assignment. All 2002 OTUs were identified at bacterial genus level, with 198 species names assigned, and others were identified to higher taxonomical hierarchies. The identified ASVs and OTUs were subsequently combined, and taxa assigned to non-Bacteria, Cyanobacteria, Chloroplast, and Rickettsiales were removed from the dataset. The bacterial dataset agglomerated at the genus level yielded a new dataset covering 1927 taxa across 40 samples with singletons and doubletons removed.

Similarly, 3,232,676 raw reads for eukaryotes were obtained from 40 samples by sequencing ITS1 region of fungal ribosomal RNA. After quality control, denosing, and removal of chimera sequences, 1,704,072 high-quality sequences and 14,801 ASVs were obtained. Out of this, 1747 ASVs were assigned to 143 fungal species. Meanwhile, 13,054 ASVs, which could not be identified at the species level, were clustered into 719 OTUs at 97% identity level and were again subjected to taxonomy assignment. Twelve OTUs were identified at fungal species level, with 7 species names assigned, and others were identified to higher taxonomical hierarchies. The identified ASVs and OTUs were subsequently combined, and non-Fungi and plant taxa were removed from the dataset. The dataset agglomerated at the species level yielded a new dataset covering 843 taxa across 40 samples with singletons and doubletons removed.

### 3.2. Shifting Taxon Composition at High Hierarchy

**Figure 1:**
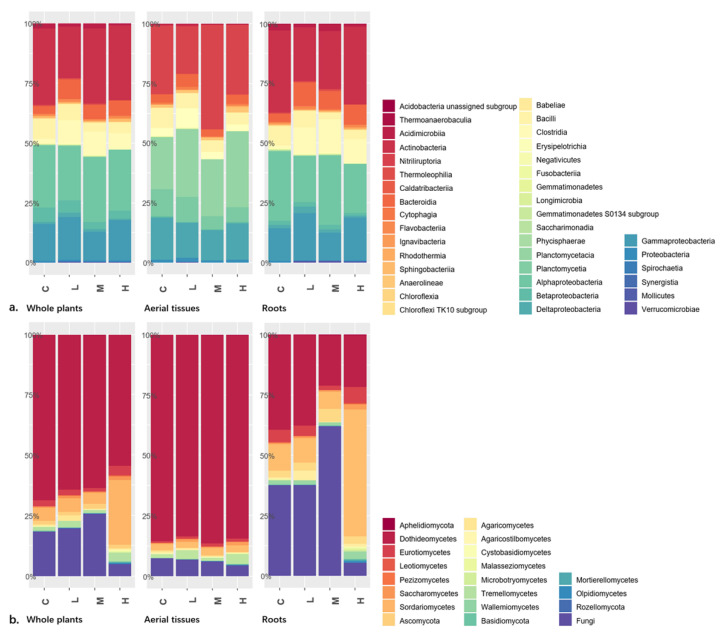
The community composition of endophytes at class or higher levels in whole plants, and aerial tissues or roots separately: (**a**), the composition of bacterial classes which were relatively even between different tissues; (**b**) the composition of fungal classes which showed dramatic difference between aerial tissues and roots. C, L, M, and H in x-axis represented control, low, medium, and high level ionizing radiation treatments, respectively.

Comparing plant tissue types or radiation levels, our results indicated that the composition dissimilarity at class level was higher in fungal communities than in bacterial communities. Class Actinobacteria (mostly Actinomycetales) followed by classes Alphaproteobacteria (mostly Rhizobiales and Sphingomonadales) and Gammaproteobacteria (mostly Xanthomonadales and Oceanospirillales) dominated the endophytic bacterial communities in aerial tissues, roots, and the whole plant of *K. schrenkianum* (Figure 1a). No bacterial class showed significant difference in abundance with radiation level.

Dothideomycetes, Sordariomycetes, and unassigned fungi were the most prevalent fungal groups. Dothideomycetes was the most abundant class in the endophytic fungal communities associated with *K. schrenkianum* (Figure 1b). Dothideomycetes significantly predominated the aerial tissues regardless of the radiation level while it was the second most abundant fungal class in the roots (Table S1). Order Pleosporales dominated Dothideomycetes class. Unassigned fungi were the most abundant group in the roots of plants from control, low, and medium radioactive environments while Sordariomycetes (mostly Hypocreales, Microascales, Sordariales, and Xylariales) was the predominant fungal class in the roots of plants from high radioactive environment.

Among the endophytic fungi of *K. schrenkianum* roots, the abundance of ascomycetous classes Leotiomycetes, Sordariomycetes, and unassigned fungi significantly varied among the radiation levels (Table S1). Meanwhile, among the endophytic fungi from aerial tissues, the abundance of basidiomycetous class Agaricomycetes and unassigned fungi significantly varied among the radiation levels. In the whole plant, abundance of the members of classes Leotiomycetes, Sordariomycetes, and Mortierellomycetes and that of the members of phyla Aphelidiomycota and Rozellomycota significantly differed among the radiation levels. In summary, endophytic fungi showed shifting community composition among treatments at a high taxonomical hierarchy according to present results.

### 3.3. Richness of Endophytes

**Figure 2:**
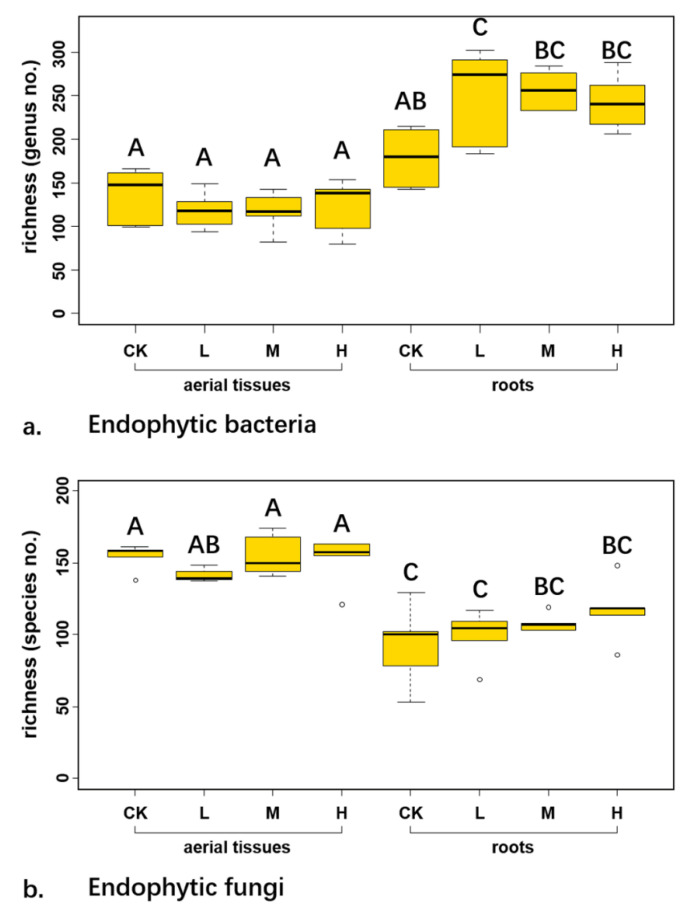
The genus richness of endophytic bacterial (**a**) and species richness of endophytic fungal (**b**) communities in each treatment. C, L, M, and H in x-axis represented control, low, medium, and high level ionizing radiation treatments, respectively. Capitalized letters showed significance at *p* = 0.05 level in HSD test.

The average taxon richness at genus level of endophytic bacteria across control and three radiation levels was 163.60 ± 27.95 in aerial tissues, while it was 252.90 ± 49.15 in roots (Figure 2a). The bacterial richness was significantly less in aboveground parts than in belowground parts (df = 30.122, *p* < 0.001). Two-way ANOVA showed that both the tissue types (*p* < 0.001) and its interaction with radiation level (*p* = 0.029) significantly affected the richness of endophytic bacteria. Meanwhile, the species richness of endophytic fungi across control and three radiation levels was 127.30 ± 29.21 (Figure 2b). Richness of endophytic fungi in aerial tissues was 150.60 ± 12.90 and in roots was 104 ± 21.01. Two-way ANOVA indicated that tissue origin significantly influenced the richness of endophytic fungi (*p* < 0.001). T-test showed that the fungal richness was significantly higher in aerial tissues than in roots (df = 31.543, *p* < 0.001).

The richness of endophytic bacteria and fungi in aerial tissues was similar among the control and three radiation levels (Figure 2). In addition, the richness of endophytic fungi in roots was the same among the different radiation levels while the richness of endophytic bacteria at low radiation level was significantly higher than that in control (Figure 2).

### 3.4. Community Composition among Treatments

**Figure 3:**
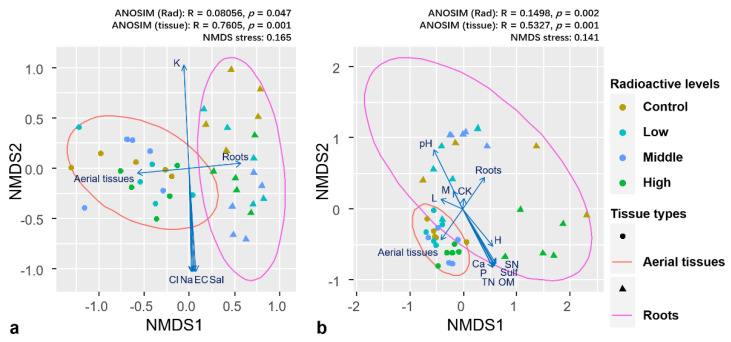
NMDS ordination indicated the difference in composition of bacterial (**a**) and fungal (**b**) communities between aerial tissues and roots, and across control and three radiation levels. ANOSIM analysis indicated that both bacterial and fungal communities were significantly different when grouped no matter with radiation levels or with tissue types. Arrows indicated environmental factors and vectors fit to NDMS ordination with significance at 0.05 level. C, L, M, and H at arrows represented control, low, medium, and high level ionizing radiation treatments, respectively. Abbreviations for environmental features: EC, electrical conductivity; OM, organic matter; TN, total nitrogen; SN, soluble nitrogen; P, available phosphorus; K, available potassium; Sal, salt; Cl, chloride ion; Sulf, sulfate; Ca, calcium ion; Na, sodium ion.

Current results indicated significant difference in community composition between two tissue types and across control and treatments. NMDS ordination revealed differences in both bacterial and fungal community compositions between aerial tissues and roots (Figure 3a,b). Environmental factors fit to NMDS indicated that the composition of the overall endophytic bacterial community was significantly affected by tissue type (R^2^ = 0.594, *p* = 0.001), yet not by radioactive level (R^2^ = 0.072, *p* = 0.448). Overall fungal community was significantly affected by both tissue type (R^2^ = 0.360, *p* = 0.001) and radioactive level (R^2^ = 0.223, *p* = 0.006). When subjected to microbial communities in different tissues separately, environmental factors fit indicated that endophyte community in aerial tissues of both bacteria and fungi were not significantly affected by radioactive level (R^2^ = 0.121, *p* = 0.609; R^2^ = 0.260, *p* = 0.126). In contrast, root endophyte community of both bacteria and fungi showed significant effect to radioactive level (R^2^ = 0.501, *p* = 0.001; R^2^ = 0.531, *p* = 0.002). ANOSIM results showed larger differences in community composition among the groups based on tissue type compared with those based on radiation level for both bacterial and fungal endophytes. We further investigated the community composition of endophytes in different tissues of *K. schrenkianum*. Bacterial communities in roots (ANOSIM: R = 0.431, *p* = 0.001) and fungal communities in both aerial tissues (ANOSIM: R = 0.2813, *p* = 0.005) and roots (ANOSIM: R = 0.3903, *p* = 0.001) showed significant differences in composition among the radiation levels. Meanwhile, the bacterial communities in aerial tissues showed no significant differences in composition among the radiation levels (ANOSIM: R = 0.1243, *p* = 0.085). In addition, PerMANOVA indicated that the community composition of endophytic bacteria and fungi was significantly affected by tissue type (F_1, 39_ = 12.021, R^2^ = 0.220, *p* = 0.001; F_1, 39_ = 15.304, R^2^ = 0.242, *p* = 0.001), radioactive level (F_3, 39_ = 2.022, R^2^ = 0.111, *p* = 0.001; F_3, 39_ = 3.143, R^2^ = 0.149, *p* = 0.001), and their interaction (F_3, 39_ = 1.534, R^2^ = 0.084, *p* = 0.032; F_3, 39_ = 2.155, R^2^ = 0.102, *p* = 0.002). PerMANOVA subjected to microbial communities in different tissues separately indicated that the composition was affected by radiation level in case of bacterial communities in aerial tissues (F_3, 19_ = 1.361, R^2^ = 0.203, *p* = 0.094) and roots (F_3, 19_ = 2.164, R^2^ = 0.289, *p* = 0.002) and fungal communities in aerial tissues (F_3, 19_ = 2.189, R^2^ = 0.291, *p* = 0.001) and roots (F_3, 19_ = 2.885, R^2^ = 0.351, *p* = 0.001). Therefore, we can conclude that sedimentary radionuclide in environment mainly influenced the root endophyte community.

### 3.5. Correlation Network

**Figure 4:**
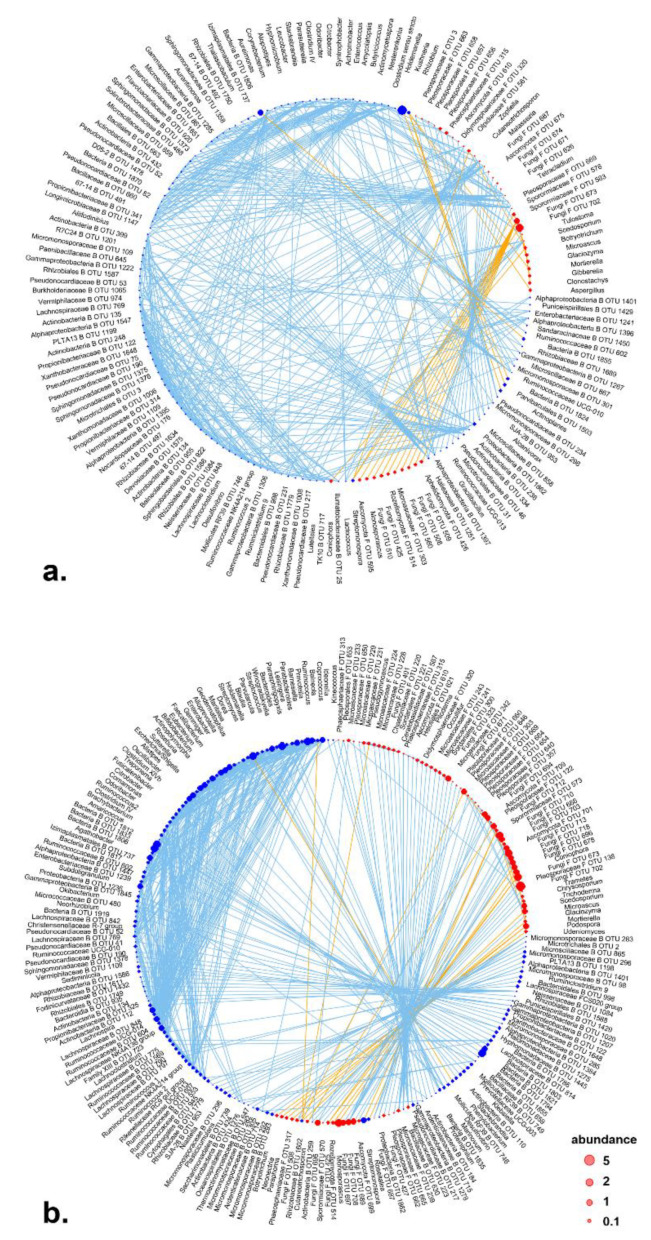
Co-occurrence network generated with endophytic bacterial and fungal genera generated at genus level for community of aerial tissues (**a**) and community of roots (**b**). Blue vertices indicate bacterial genera, red fungal genera, sky blue edges indicate positive correlations, and orange edges indicate negative correlations. Abundance was represented with normalized read counts using Hellinger transformation. All correlations were significant at *p* < 0.001 level, and with *rho* values in top 10% (for positive correlations) and bottom 10% (for negative ones) out of all *rho* values among correlations with statistical significance.

Correlation network analysis indicated that positive correlations occurred within bacterial group or within fungal group in microbiota of aerial parts and roots. In aerial tissues, positive correlations were observed among endophytic bacterial genera, while negative correlations occurred among fungal genera (Figure 4a). For example, unidentified fungal OTU_673 and OTU_702 showed strong negative correlations to several fungal taxa. Meanwhile, positive correlations could be also observed between bacteria or fungi. In roots, bacterial or fungal members in the community were more connected (Figure 4b). Concatenated negative correlations were observed between bacteria or fungi, which suggested intensive antagonism between bacteria and fungi in roots. These findings indicate denser inter-kingdom interactions in the roots rather than in the aerial tissues.

Network analysis of endophytic bacterial communities suggested a minor effect of radiation on the topology of endophytic bacterial network. The network of endophytic bacterial communities in aerial tissues showed similar connectance and modularity across control and three radioactive treatments (Table 2). Meanwhile, the root bacterial communities showed higher connectance in control and similar modularity in control and radioactive environments. Simultaneously, radiation showed a strong effect on the topology of endophytic fungal correlation network. Higher modularity and lower connectance were observed in all radiation levels compared to control regardless of the tissue type (aerial tissues or roots) (Table 2).

### 3.6. Genetic Diversity

**Figure 5:**
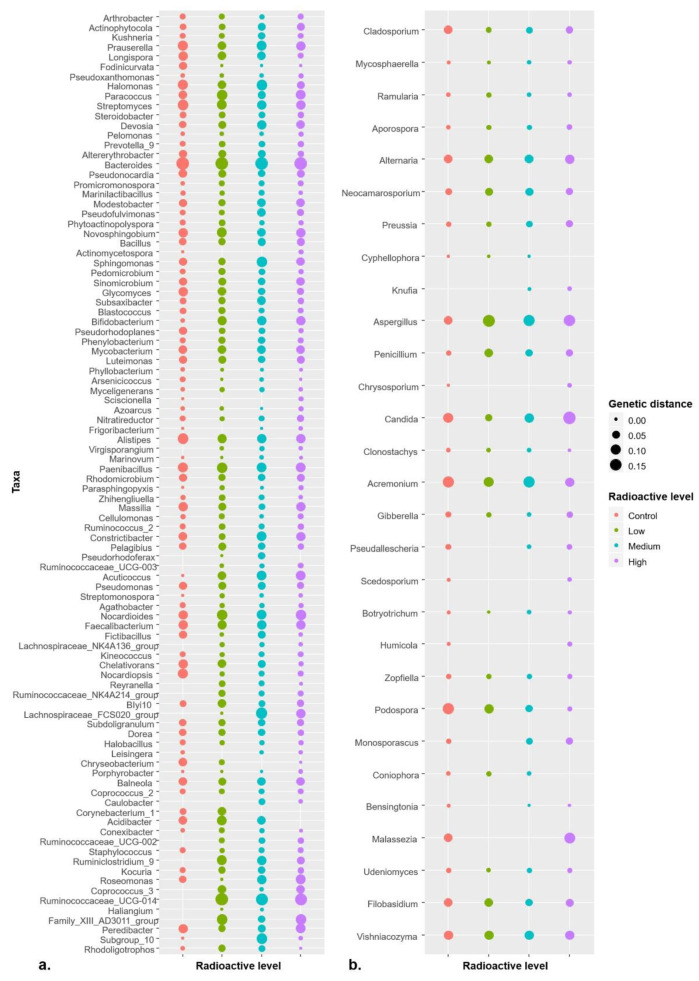
The shifting average genetic distances of bacterial (**a**) and fungal (**b**) genera among treatments of different radiation levels. Taxa shown in figure were those with significant difference in genetic distances among radiation levels tested with ANOVA. For entire list of bacterial and fungal genera with detailed values, please see Tables S2 and S3, respectively.

Pairwise distances that are calculated based on “K80” model among all ASVs available in a certain genus were used to evaluate the genetic diversity of microbial population in present study. The results showed that genetic diversity differed within control and different radiation levels for both bacterial and fungal genera. Significant differences were observed in the genetic diversity of 51 bacterial genera and 29 fungal genera (Figure 5; Table S2 for all bacterial and fungal genera proceeded to one-way ANOVA). Intriguingly, the average genetic distance among more than half of the genera in both bacterial and fungal communities was more in the radioactive environments than that in control (Figure 5; Table S2 for bacterial genera and Table S3 for fungal genera). Meanwhile, the overall genetic diversity at genus level of both bacterial and fungi in whole plant were higher in the radioactive environments than those in control treatments tested with one-way ANOVA (Table S4).

### 3.7. Effects of Environmental Factors on Endophyte Abundance or Richness

GLM was used to assess the effect of different environmental factors on endophyte abundance or richness at the bacterial genus level or fungal species level. The results showed that richness of bacterial genus negatively correlated with pH and TN in the bacterial communities of whole plant and roots (Table 3). The richness of fungal taxa for the whole plant fungal communities negatively correlated with roots. The fungal communities in roots positively correlated with TN and negatively correlated with OM. Nevertheless, there was no significant correlation among endophyte diversity and environmental factors in aerial tissues, that neither bacterial genera richness nor fungal species richness showed significant correlation to environment factors.

Endophytic taxa with significant preference to certain niches or environmental factors were screened at different taxonomical hierarchies with GLM (Table 4). The abundance of bacterial genera Cupriavidus and Brevibacterium positively correlated with P and OM in whole plant bacterial community (Table 4). The abundance of fungal taxa Saitozyma podzolica, Sporormiaceae FOTU_583, Aporospora FOTU_199, fungal genus Saitozyma and Neocamarosporium, and class Dothideomycetes positively correlated with roots. No fungal group showed significant correlation with abiotic environmental factors except Acremonium chrysogenum, which negatively correlated with medium radiation level.

## 4. Discussion

Studies have reported differences in endophytic communities between root and aerial tissues in various plants [46,47,48]. In addition to the difference in biochemical environments between roots and aerial tissues, distinct environmental propagule pools above ground and underground, colonizing and coexisting mechanisms with roots and aerial tissues, and inter-tissue transport limitation contribute to the differences in communities [9,46,49]. Community composition in *K. schrenkianum* was different between aerial tissues and roots for both bacteria and fungi. Interestingly, the diversity of endophytic bacteria in aerial tissues was less than that in roots, whereas the diversity of endophytic fungi showed an opposite pattern. Microbial species in soil are generally more than that in air. Therefore, the bacterial communities might have largely assembled by immigrating from propagules from the environmental source. However, a differential selection by the tissue significantly shaped the fungal communities in present study. The community composition of endophytic fungi determined by the host is restricted by the environmental propagule pool. For example, soil related taxa, including *Saitozyma podzolica* and Sporormiaceae_F_OTU_583, showed significant preference for the root in *K. schrenkianum* in multiple stepwise regression model (Table 3). They would be representatives of soil borne fungi as *S*. *podzolica* (previously *Candida podzolica*) was first identified as a soil yeast [50,51], and Sporormiaceae members are famous saprobes cosmopolitan to various substrate including soil [52]. These soil inhabiting microbes might be able to colonized *K*. *schrenkianum* and switched their life styles between endophytism and saprophytism [53,54]. In summary, our research suggested that the assembly of both bacterial and fungal endophytic microbiota in roots of *K. schrenkianum* were affected by soil microbial propagule pools, while roots have stronger selection to fungal colonizer.

In addition to the differences in community composition, our study demonstrates differences between aerial tissues and roots in the responses of endophytic microbiota to environmental factors. The diversity of endophytic microbiota in roots correlated with soil chemical characteristics, while the microbiota in aerial tissues showed no response to varied environmental factors. We observed increased diversity in endophytes associated with root from control treatment to radioactive environments and not in endophytes associated with aerial tissues. These findings indicate a larger effect of environment factors on root endophytic microbiota.

Soil pH is a major determinant of microbial community structure and assembly [55,56,57]. Lauber, Hamady, Knight and Fierer [55] stated that soil pH predicted the composition of soil bacterial communities, and phylogenetic diversity attained a peak at near neutral pH. Diversity of endophytic bacteria showed a significant negative correlation with soil pH of our study sites that ranged from 8.8 to 9.6. This is consistent with the findings of Lauber, Hamady, Knight and Fierer [55]. Conclusively, the recruitment of root bacterial microbiota is largely dependent on the diversity of soil species. However, we found no correlation between pH and fungal diversity. Rousk et al. had suggested that fungi prefer a wider pH range for optimal growth compared with bacteria Rousk, Bååth, Brookes, Lauber, Lozupone, Caporaso, Knight and Fierer [56].

In a recent study, Bahram, et al. [58] reported strong antagonism between fungi and bacteria globally. In *K. schrenkianum*, we found limited interaction between bacteria and fungi; however, there was bacterial-fungal competition at the niche level. The co-occurrence correlations are prone to restrict to the intra-kingdom members of bacteria and fungi. Meanwhile, inter-kingdom members were negatively correlated. In the roots, bacteria and fungi were less correlated, which indicates less competition in the rhizosphere.

Despite the presence of various chemical factors of soil, radiation was the dominant factor that structured the endophytic communities. Higher genetic diversity was found in radioactive environments for both bacterial and fungal communities. However, mutations arising from ionizing radiation and its effect on genetic diversity need to be investigated. Environmental stress resulted in increase in genetic diversity in the community. Nevo [59] discovered higher genetic diversity in several tested model organisms under stressful environments with thermal, chemical, climatic, and biotic stresses. Increase in genetic diversity enhanced probability of population survival [60], and populations with low genetic diversity had reduced fitness and increased extinction rates [61]. Therefore, higher genetic diversity acts as an inherent mechanism of community assembly to maintain the community under moderately stressed environments.

Meanwhile, the current study also revealed increased community diversity in roots at radiation stressed treatments apart from the increased population genetic diversity. Diversity as an essential descriptive and metrological feature, could predict the stability, function, and productivity of an ecosystem [62,63,64]. High species and phylogenetic diversity are related to high functional diversity and redundancy. However, it is difficult to establish direct correspondence yet, because studies that convincingly tested the linkage between phylogeny and physiology in microbial communities are limited [62]. High diversity of endophytic communities in *K. schrenkianum* roots might imply that the radionuclide sediments in soil severely stressed the root niche and consequently diverse microbes assembled to maintain a stable endophytic microbial communities.

Environmental radiation evoked different responses in bacterial and fungal communities. Vries, et al. [65] showed that bacterial networks were less stable under drought stress than fungal networks in grassland mesocosms set in UK. However, in the present study, the community structure of endophytic fungi was sensitive to radiation more than that of endophytic bacteria. Fungal co-occurrence networks were more fragmented under radiation-stressed environments; connectance decreased and modularity increased at all radiation levels. We observed significant differences in the community composition of fungi in both aerial tissues and roots and of bacteria in roots across all treatments. In addition, the fungal community composition showed dramatic differences at class level and at species level across treatments.

The current study is the first to investigate the community structure of plant symbiotic microbiota under radioactive stress. Nevertheless, present study gave additional questions when answering to mechanisms of community assembly and maintenance under the extreme environments. We did not investigate the correlation between ionizing radiation-induced mutation and increase in genetic diversity, and the inherent mechanism that drives community assembly under radiation-stressed environment. Besides, we did not analyze the OTUs identified as unassigned fungi, which showed extraordinary abundance and dominance in roots. The OTUs matched (low E-value and identity) the members of phylum Ascomycota (data not shown). However, these fungi are still unknown (since no strain was recovered from the plant). Further studies are necessary to expand our knowledge to plant-microbe symbiosis under radioactive environments.

## 5. Conclusions

The current study is the first to investigate the community structure of plant symbiotic microbiota under radioactive stress. Actinobacteria and Dothideomycetes predominated the bacterial, and fungal endophytic communities of *K. schrenkianum*, respectively. More diverse fungi inhabited aerial tissues than roots while it was contrary for bacteria. Our results showed that environmental radiation affect endophytic communities in a mild way when it is within tolerable range for organisms. Radiation levels affected the community composition of endophyte communities and co-occurrence networks. The genetic diversity of both endophytic bacteria and fungi at genus level were generally higher in radioactive habitats. Nevertheless, geochemical factors determined root bacterial community diversity rather than radiation level. The present study revealed our knowledge limitation on ecological interaction under radioactive environments as well. Thus future studies are necessary to expand our knowledge to the biodiversity, maintenance mechanism of population genetic diversity, and the inter-kingdom interaction involved in plant-microbe symbiosis under radioactive environments.

## Figures and Tables

**Table 1:** Radiation levels and geochemical characteristics of sampling sites.

Sampling Sites	Site 1	Site 2	Site 3	Site 4
Radiation level	control	low	medium	high
pH	9.6	9.4	9.6	8.8
organic matter (g/kg)	9.4	8.8	7.2	12.3
total nitrogen (g/kg)	0.84	0.83	0.68	1.26
soluble nitrogen (mg/kg)	52	34.8	39.8	155
available phosphorus (mg/kg)	7.2	11	3.8	29.4
available potassium (mg/kg)	580	464	350	417
salt content (g/kg)	3	4.8	5	4.8
electrical conductivity (mS/cm)	1122	1610	1894	1610
chloride ion (g/kg)	1.1	1.4	2.3	1.6
Sulfate (g/kg)	0.03	0.03	0.03	0.42
Calcium ion (g/kg)	0.11	0.15	0.11	0.3
Magnesium ion (g/kg)	0.05	0.08	0.05	0.08
Sodium ion (g/kg)	1.01	1.56	1.94	1.32

**Table 2:** Network characteristics of bacterial and fungal co-occurrence networks in aerial tissues and roots.

	All Nodes	Connected Nodes	Connection	Connectance	Modularity
**Bacterial Community in Aerial Tissues**					
Control	319	294	3405	0.079	0.730
Low	291	275	2466	0.065	0.816
Middle	269	242	1746	0.060	0.823
High	280	254	2396	0.075	0.730
**Bacterial Community in Roots**					
Control	426	386	4610	0.062	0.802
Low	498	459	4229	0.040	0.791
Middle	473	438	3421	0.036	0.824
High	462	426	2966	0.033	0.859
**Fungal Community in Aerial Tissues**					
Control	125	91	703	0.172	0.195
Low	97	67	90	0.041	0.831
Middle	144	111	293	0.048	0.824
High	129	93	184	0.043	0.816
**Fungal Community in Roots**					
Control	123	101	556	0.110	0.456
Low	98	75	133	0.048	0.859
Middle	128	95	263	0.059	0.875
High	139	105	372	0.068	0.709

**Table 3:** Multiple stepwise regression model results for genus (for bacteria) or species (for fungi) richness that significantly correlated with tissue type or environmental factors (TN: total nitrogen; OM: organic matter).

Explanatory Variable	T	*p*-Value
**Whole Plant Bacterial Community**		
tissue (root)	25.41	<0.001
pH	−5.68	<0.001
TN	−5.40	<0.001
**Root Bacterial Community**		
pH	−9.06	<0.001
TN	−8.75	<0.001
**Whole Plant Fungal Community**		
tissue (root)	−12.99	<0.001
**Root Fungal Community**		
OM	−3.09	0.015
TN	3.43	0.007

**Table 4:** Multiple stepwise regression model results for bacterial and fungal taxa that significantly correlated with tissue type or environmental factors (P: available phosphorus; OM: organic matter).

Taxon	Explanatory Variable	T	*p*-Value
**Whole Plant Bacterial Community (Genus)**			
*Cupriavidus*	P	34.12666	<0.001
*Brevibacterium*	OM	5.984721	<0.001
**Whole Plant Fungal Community (Species)**			
*Saitozyma podzolica*	tissue (root)	326.7589	<0.001
Sporormiaceae_F_OTU_583	tissue (root)	65.27728	<0.001
*Aporospora*_F_OTU_199	tissue (root)	−5.26803	<0.001
**Whole Plant Fungal Community (Genus)**			
*Saitozyma*	tissue (root)	326.7589	<0.001
*Neocamarosporium*	tissue (root)	−5.98923	<0.001
**Whole Plant Fungal Community (Class)**			
Dothideomycetes	tissue (root)	−6.271	<0.001
**Aerial Tissue Fungal Community (Species)**			
*Acremonium chrysogenum*	RadM	−40.1085	<0.001

## Data Availability

The ASV and OTU sequences have been deposited in GenBank of National Center for Biotechnology Information under the accession numbers PRJNA613570 (bacterial 16S sequences) and MT351182-MT353643 (fungal ITS1 sequences) and the raw sequences in the Sequence Read Archive of NCBI under BioProject PRJNA625640 (for bacterial data) and PRJNA613597 (for fungal data).

## Supplementary Materials

The following are available online at https://www.mdpi.com/article/10.3390/microorganisms9051050/s1, Figure S1: A schematic diagram for OTU clustering and taxonomy assignments. Table S1: One-way ANOVA showed significant abundance shift among radioactive levels in some fungal classes. Table S2: Average pairwise distances of sequences indicating different genetic diversities for each bacterial genus, and the significance of difference tested with one-way ANOVA among radioactive levels. NA values means there was no or only 1 ASV detected at the treatment for the genus, and thus cannot be calculated pairwise distances. If a genus showed NA in all four treatments, the genus is removed from the table for concise reason. Table S3: Average pairwise distances of sequences indicating different genetic diversities for each fungal genus, and the significance of difference tested with one-way ANOVA among radioactive levels. NA values means there was no or only 1 ASV detected at the treatment for the genus, and thus cannot be calculated pairwise distances. If a genus showed NA in all four treatments, the genus is removed from the table for concise reason. Table S4: One-way ANOVA results shows different genetic diversity at genus level among treatments for both bacteria and fungi.
